# Diabetes mellitus and chronic kidney disease in the Eastern Mediterranean Region: findings from the Global Burden of Disease 2015 study

**DOI:** 10.1007/s00038-017-1014-1

**Published:** 2017-08-03

**Authors:** GBD 2015 Eastern Mediterranean Region Diabetes and Chronic Kidney Disease Collaborators, Maziar Moradi-Lakeh, Charbel El Bcheraoui, Ibrahim Khalil, Raghid Charara, Ashkan Afshin, Haidong Wang, Michael Collison, Kristopher J. Krohn, Adrienne Chew, Farah Daoud, Christopher D. Blosser, Leslie Cornaby, Kyle J. Foreman, Nicholas J. Kassebaum, Laura Kemmer, Michael Kutz, Patrick Liu, Ben Zipkin, Johan Ärnlöv, Kalkidan Hassen Abate, Alireza Ahmadi, Hamid Ahmadieh, Muktar Beshir Ahmed, Ziyad Al-Aly, Khurshid Alam, Deena Alasfoor, Raghib Ali, Reza Alizadeh-Navaei, Juma M. Alkaabi, Ala’a Alkerwi, Rajaa Al-Raddadi, Khalid A. Altirkawi, Nelson Alvis-Guzman, Erfan Amini, Nahla Anber, Palwasha Anwari, Solomon Weldegebreal Asgedom, Tesfay Mehari Atey, Leticia Avila-Burgos, Ashish Awasthi, Peter Azzopardi, Till Bärnighausen, Umar Bacha, Aleksandra Barac, Shahrzad Bazargan-Hejazi, Neeraj Bedi, Derbew Fikadu Berhe, Addisu Shunu Beyene, Zulfiqar A. Bhutta, Boris Bikbov, Mulugeta M. Birhanu, Zahid A. Butt, Lucero Cahuana-Hurtado, David O. Carpenter, Juan Jesus Carrero, Jee-Young Jasmine/J Choi, Hadi Danawi, Samath D. Dharmaratne, Eric L. Ding, Shirin Djalalinia, Kerrie E. Doyle, Hedyeh Ebrahimi, Aman Yesuf Endries, Alireza Esteghamati, Maryam S. Farvid, Seyed-Mohammad Fereshtehnejad, Tesfaye Regassa Feyissa, Florian Fischer, Tsegaye Tewelde Gebrehiwot, Philimon N Gona, Sameer Vali Gopalani, Masako Horino, Mohamed Hsairi, Mihajlo B. Jonas, Aida Jimenez-Corona, Denny John, Jost B. Jonas, Amir Kasaeian, Andre Pascal Kengne, Ezra Belay Khan, Daniel Kim, Yun Jin Kim, Yohannes Kinfu, Katarzyna A. Kissimova-Skarbek, Ai Koyanagi, Heidi J. Larson, Anders Larsson, Yongmei Li, Paulo A. Lotufo, Raimundas Lunevicius, Azeem Majeed, Reza Malekzadeh, Deborah Carvalho Malta, Mohsen Mazidi, Ziad A. Memish, Walter Mendoza, Mubarek Abera Mengistie, George A. Mensah, Haftay Berhane Mezgebe, Ted R. Miller, Muktar Sano Kedir Mohammed, Shafiu Mohammed, Ulrich O. Mueller, Gabriele Nagel, Cuong Tat Nguyen, Quyen Le Nguyen, Vuong Minh Nong, Jean Jacques N. Noubiap, Felix Akpojene Ogbo, Alberto Ortiz, Erika Ota, Tejas Patel, Jonathan Pearson-Stuttard, Norberto Perico, Max Petzold, Farhad Pishgar, Farshad Pourmalek, Mostafa Qorbani, Vafa Rahimi-Movaghar, Rajesh Kumar Rai, Saleem M. Rana, David Laith Rawaf, Salman Rawaf, Giuseppe Remuzzi, Andre M. N. N. Renzaho, Satar Rezaei, Gholamreza Roshandel, Dietrich Rothenbacher, Mahdi Safdarian, Sare Safi, Saeid Safiri, Mohammad Ali Sahraian, Payman Salamati, Abdallah M. Samy, Juan Ramon Sanabria, Maria Dolores Sanchez-Niño, Milena M. Santric Milicevic, Benn Sartorius, Sadaf G Sepanlou, Masood Ali Shaikh, Diego Augusto Santos Silva, Dayane Gabriele Alves Silveira, Badr H. A. Sobaih, Rizwan Suliankatchi Abdulkader, Rafael Tabarés-Seisdedos, Arash Tehrani-Banihashemi, Mohamad-Hani Temsah, Roman Topor-Madry, Bach Xuan Tran, Kingsley Nnanna Ukwaja, Olalekan A. Uthman, Job F. M. van Boven, Tolassa Wakayo, Andrea Werdecker, Abdulhalik Workicho, Mohsen Yaghoubi, Yuichiro Yano, Mehdi Yaseri, Naohiro Yonemoto, Mustafa Z. Younis, Anthony Lin Zhang, Aisha O. Jumaan, Theo Vos, Mohsen Naghavi, Simon I. Hay, Christopher J. L. Murray, Ali H. Mokdad

**Affiliations:** Institute for Health Metrics and Evaluation, University of Washington, Seattle, WA USA

**Keywords:** Diabetes, Chronic kidney disease, Burden of disease, Eastern Mediterranean Region

## Abstract

**Objectives:**

We used findings from the Global Burden of Disease 2015 study to update our previous publication on the burden of diabetes and chronic kidney disease due to diabetes (CKD-DM) during 1990–2015.

**Methods:**

We extracted GBD 2015 estimates for prevalence, mortality, and disability-adjusted life years (DALYs) of diabetes (including burden of low vision due to diabetes, neuropathy, and amputations and CKD-DM for 22 countries of the EMR from the GBD visualization tools.

**Results:**

In 2015, 135,230 (95% UI 123,034–148,184) individuals died from diabetes and 16,470 (95% UI 13,977–18,961) from CKD-DM, 216 and 179% increases, respectively, compared to 1990. The total number of people with diabetes was 42.3 million (95% UI 38.6–46.4 million) in 2015. DALY rates of diabetes in 2015 were significantly higher than the expected rates based on Socio-demographic Index (SDI).

**Conclusions:**

Our study showed a large and increasing burden of diabetes in the region. There is an urgency in dealing with diabetes and its consequences, and these efforts should be at the forefront of health prevention and promotion.

**Electronic supplementary material:**

The online version of this article (doi:10.1007/s00038-017-1014-1) contains supplementary material, which is available to authorized users.

## Introduction

Diabetes is an important cause of disability and death around the world and is a major risk factor for other diseases (GBD 2015 DALYs/HALE Collaborators 2016; Moradi-Lakeh et al. 2016b). The World Health Organization Eastern Mediterranean Region (EMR) has the highest age-standardized rate of disability-adjusted life years (DALYs) from diabetes (GBD 2015 DALYs/HALE Collaborators 2016; Institute for Health Metrics and Evaluation 2016). Analysis of the global burden of disease (GBD) 2013 study showed that the increasing burden of diabetes in the EMR in recent decades is beyond that expected based on the demographic changes of population growth and aging, and is also due to increases in age-specific DALY rates (Mokdad et al. 2016; Moradi-Lakeh et al. 2016b). This increasing trend has been reported by other studies as well (Sozmen et al. 2015) and is mainly because of the epidemics of obesity and physical inactivity as the main risk factors for type 2 diabetes mellitus (Mokdad et al. 2014, 2016; Sozmen et al. 2015).

Tracking of personal health spending in the United States shows that diabetes imposes the highest health care spending (Dieleman et al. 2016). International Diabetes Federation estimated US $17.1–27.7 billion is spent annually in the Middle East and North Africa on diabetes, an amount which is expected to double by 2040 (IDF 2015). In this report, we present estimates of the burden of diabetes mellitus and chronic kidney disease due to diabetes mellitus (CKD-DM) from the Global Burden of Disease 2015 study.

## Methods

GBD 2015 covers 195 countries, 21 regions, and seven super-regions from 1990 to 2015 for 315 diseases and injuries, 2619 sequelae, and 79 risk factors by age and sex. Detailed descriptions of GBD 2015 methodology and specific diabetes mellitus methodology have been provided elsewhere (GBD 2015 DALYs/HALE Collaborators 2016; GBD 2015 Disease and Injury Incidence and Prevalence Collaborators 2016; Duncan et al. 2017; Moradi-Lakeh et al. 2016b; GBD 2015 Causes of Death Collaborators 2016).

We evaluated the burden of diabetes and CKD-DM in 22 EMR countries: Afghanistan, Bahrain, Djibouti, Egypt, Iran, Iraq, Jordan, Kuwait, Lebanon, Libya, Morocco, Pakistan, Palestine, Qatar, Saudi Arabia, Somalia, Sudan, Syria, Tunisia, United Arab Emirates (UAE) and Yemen. The total population of the EMR is over 580 million people.

Diabetes mellitus in GBD is considered both as a disease and a metabolic risk factor. In this study, we focus on its burden as a disease. The burden of uncomplicated diabetes, vision loss caused by diabetes (moderate low vision, severe low vision, and blindness), diabetic neuropathy, diabetic foot due to neuropathy, and amputation are included in the burden of diabetes (Duncan et al. 2017; Moradi-Lakeh et al. 2016b). Also, we estimated burden of CKD-DM as part of the chronic kidney disease burden.

All-cause mortality envelopes (total number of deaths) were first estimated for each country during the period of 1990–2015. For this purpose, we used all accessible data from vital registration systems, sibling history surveys, sample registration data, and household recall of deaths. We extracted causes of death data from the same sources, as well as available verbal autopsies, and then used cause of death ensemble modeling to estimate the number of deaths from diabetes and CKD-DM by age, sex, country, and year (GBD 2015 DALYs/HALE Collaborators 2016; Duncan et al. 2017; Moradi-Lakeh et al. 2016b). In this approach, a large variety of possible models are explored to estimate trends in causes of death. Possible models are identified based on a covariate selection algorithm that yields several plausible combinations of covariates; they are then run through different model classes, including mixed effects linear models and spatiotemporal Gaussian process regression models for cause fractions and death rates. All models for each cause of death are then assessed using out-of-sample predictive validity and combined into an ensemble with optimal out-of-sample predictive performance (Foreman et al. 2012).

We updated our previous systematic review for the GBD study separately for non-fatal outcomes of diabetes mellitus and CKD-DM. Data on incidence, prevalence, and excess mortality were extracted from data sources. We assumed no remission for diabetes. Bayesian meta-regression analysis through DisMod-MR 2.1 was used for disease modeling. Model-based epidemiological estimates in combination with disability weights were used to calculate cause-specific years lived with disability (YLDs) for each age, sex, location, and year. DALYs were calculated through summation of years of life lost (YLLs) and YLDs (GBD 2015 DALYs/HALE Collaborators 2016; GBD 2015 Disease and Injury Incidence and Prevalence Collaborators 2016).

In GBD 2015, we used country-location estimates of a composite Socio-demographic Index (SDI) based on the geometric mean of income per capita, average years of schooling in individuals older than 15 years, and total fertility rate. The numbers were rescaled to a number between zero and one, based on highest and lowest country-location measures. In 2015, SDI had a range between 0.1506 (Somalia) and 0.8747 (United Arab Emirates) in the EMR. We used SDI to estimate expected burden for each disease based on the demographic and social conditions of each country in each year (GBD 2015 DALYs/HALE Collaborators 2016).

We report 95% uncertainty intervals (UI) for each estimate, including rates, numbers of deaths, and DALYs. We estimated UIs by taking 1000 samples from the posterior distribution of each quantity and using the 25th and 975th-ordered draw of the uncertainty distribution.

## Results

**Fig. 1:**
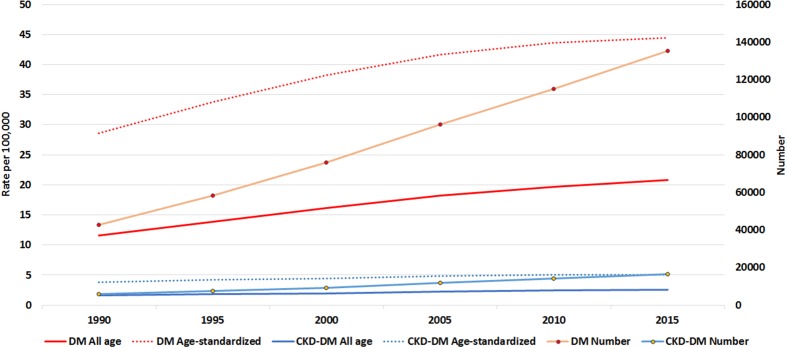
Trend of number of deaths, all-age and age-standardized mortality of diabetes mellitus (DM) and chronic kidney disease due to diabetes mellitus (CKD-DM). (Global Burden of Disease 2015 study, Eastern Mediterranean Region, 1990–2015)

In 2015, 135,230 (95% UI 123,034–148,184) individuals died from diabetes and 16,470 (95% UI 13,977–18,961) from CKD-DM in the EMR. These numbers represent 216 and 179% increases in the number of deaths due to diabetes and CKD-DM, respectively, compared to 1990. Figure 1 shows this increasing trend is not only for the number of deaths, but also for all ages and age-standardized mortality rates.

**Fig. 2:**
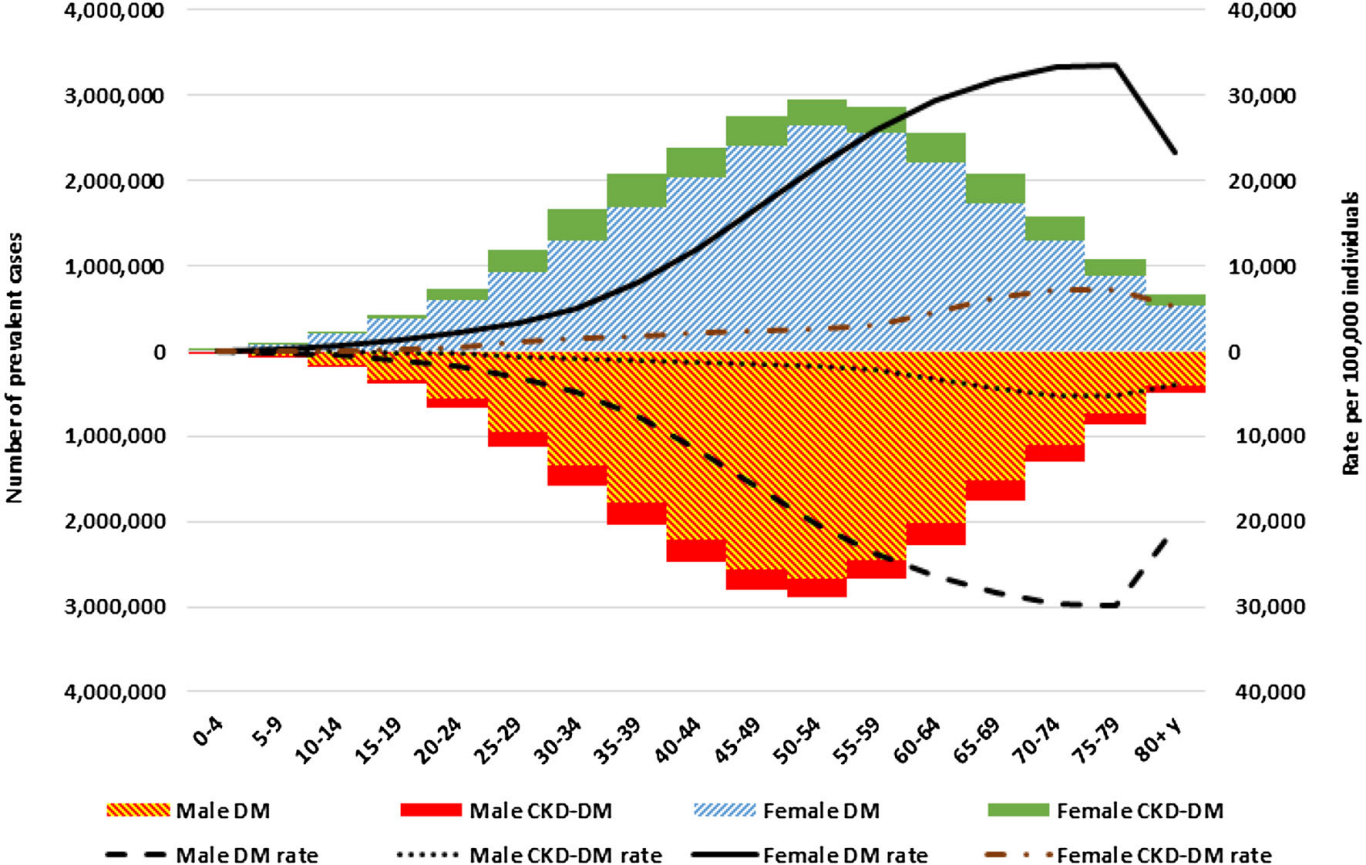
Number and rate of prevalence cases of diabetes mellitus(DM) and chronic kidney disease due to diabetes mellitus(CKD-DM) in the Eastern Mediterranean Region, 2015. (Global Burden of Disease 2015 study, Eastern Mediterranean Region, 2015)

The total number of people with diabetes in the EMR in 2015 was 42.3 million (95% UI 38.6–46.4 million). The highest prevalence rates of DM and CKD-DM were observed among those aged 70–79 years old; however, the highest numbers of cases were among the younger age groups. The patterns of prevalence were similar in both sexes (Fig. 2).

Total DALYs from diabetes were 6,708,539 (95% UI 5,451,990–8,148,834) in 2015 and 2,285,117 (95% UI 1,892,297–2,792,790) in 1990. For CKD-DM, total DALYs were 568,351 (95% UI 490,064–653,946) in 2015 and 234,194 (95% UI: 201,911–272,837) in 1990. In 2015, the proportion of YLLs to DALYs was 45% for diabetes mellitus and 73% for CKD-DM.

**Fig. 3:**
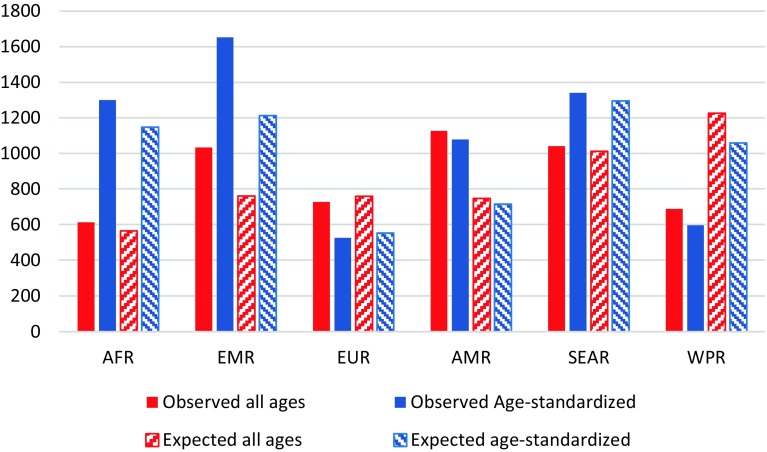
Rates of disability-adjusted life years of diabetes mellitus per 100,000 population in the World Health Organization regions. *AFR* African region, *EMR* Eastern Mediterranean region, *EUR* European region, *AMR* Region of Americas, *SEAR* Southeast Asia region, *WPR* Western Pacific region. (Global Burden of Disease 2015 study, World Health Organization regions, 2015)

**Fig. 4:**
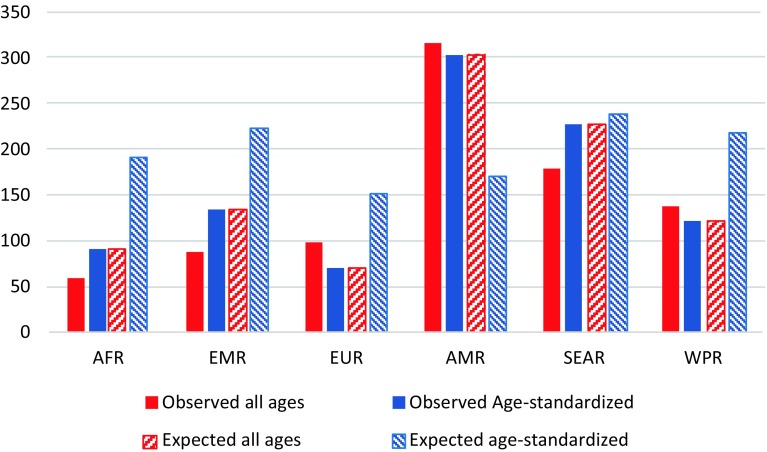
Rates of disability-adjusted life years of chronic kidney disease due to diabetes mellitus per 100,000 population in the World Health Organization regions. *AFR* African region, *EMR* Eastern Mediterranean region, *EUR* European region, *AMR* Region of Americas, *SEAR* Southeast Asia region, *WPR* Western Pacific region. (Global Burden of Disease 2015 study, World Health Organization regions, 2015)

The burden of diabetes mellitus as a percentage of total DALYs was 1.1% (95% UI 1.0–1.3%) in 1990 and increased to 2.9% (95% CI 2.6–3.3%) in 2015. These percentage were 0.11% (95% UI 0.10–0.13%) and 0.25% (95% CI 0.22–0.28%) for CKD-DM in 1990 and 2015, respectively. The age-standardized observed DALY rate of diabetes in the EMR was higher than in all other WHO regions. Also, observed DALY rates of diabetes in the EMR were higher than the expected (based on SDI) values (Fig. 3). However, observed DALY rates for CKD-DM were less than the expected rates (Fig. 4).

Table 1 lists DALY rates of diabetes and CKD-DM in each of the EMR countries in 2015. Morocco, Tunisia, and Bahrain had the highest DALY rates of diabetes, and Tunisia, Saudi Arabia, and Afghanistan had the highest DALY rates of CKD-DM.

**Table 1:** Disability-adjusted life years (DALYs) of diabetes mellitus and chronic kidney disease due to diabetes mellitus in the Eastern Mediterranean Region countries, 2015

Location	Chronic kidney disease due to diabetes mellitus						Diabetes mellitus					
	Male			Female			Male			Female		
	Rate	95% UI		Rate	95% UI		Rate	95% UI		Rate	95% UI	
Afghanistan	137	73	222	204	124	322	735	550	970	1201	874	1645
Bahrain	51	41	64	47	37	56	1569	1210	1988	1529	1188	1926
Djibouti	111	61	215	82	52	146	1359	713	2691	856	483	1642
Egypt	35	27	44	34	26	41	1265	1016	1570	1294	1029	1595
Iran	75	56	97	66	51	83	908	685	1155	925	684	1191
Iraq	25	19	32	28	21	35	1287	990	1613	1494	1157	1926
Jordan	130	104	157	113	94	135	1052	810	1343	956	715	1242
Kuwait	49	39	61	51	40	62	663	475	889	629	453	839
Lebanon	70	47	101	86	60	117	1232	923	1585	1280	932	1627
Libya	117	79	166	115	84	154	865	655	1112	1032	772	1334
Morocco	122	85	174	124	87	168	1663	1265	2118	2061	1548	2611
Oman	70	54	86	80	66	95	1203	916	1525	1168	888	1483
Pakistan	114	86	141	67	54	81	895	699	1109	1091	847	1371
Palestine	51	39	65	42	34	52	572	432	733	547	414	707
Qatar	41	31	52	41	31	52	1015	748	1325	1077	801	1407
Saudi Arabia	226	164	266	159	140	181	655	472	876	514	371	686
Somalia	79	43	154	75	41	142	657	328	1303	527	265	1021
Sudan	89	53	150	85	52	131	689	515	909	787	590	1013
Syria	24	17	33	22	16	29	510	381	672	578	423	764
Tunisia	264	196	354	183	137	238	1783	1396	2210	1527	1170	1920
UAE	119	72	186	65	46	91	1231	890	1630	916	667	1196
Yemen	86	50	143	107	62	184	536	386	719	792	571	1102

Global Burden of Disease 2015 study, Eastern Mediterranean Countries, 2015

## Discussion

Our study showed that the burden of diabetes has increased considerably during the last 25 years in the EMR. This burden is higher than expected based on the demographic and social status of the countries in the region. Clearly, the region’s health systems have not performed at the expected level, given their socio-demographic status, to control and prevent diabetes and CKD. This is in contrast to the European region and Western Pacific region, where observed levels are lower than expected levels. There are several potential reasons for such differenced: people in the EMR have lower perceived risk, and access to and quality of health care are lesser in this region (Mokdad et al. 2014; Moradi-Lakeh et al. 2016b) Our results call for urgent efforts to address the burden of diabetes in the region.

Several interventions have been suggested for prevention and control of diabetes. Although the effectiveness and cost-effectiveness of all interventions are not promising, there is evidence of several successful experiences around the world (Davies et al. 2017; Sun et al. 2017). For example, the National Diabetes Prevention Program showed successful changes in determinants of diabetes (Ely et al. 2017). Indeed, a multi-sectorial approach is needed to control and prevent diabetes in the region. WHO suggests the Package of Essential Non-communicable (PEN) Disease Interventions to be used in primary health care in low-resource settings. PEN is a prioritized set of cost-effective interventions, tools, and aids to deliver an acceptable quality of care in the primary health care setting. Such interventions are feasible for adoption by most counties in the region (Zhang et al. 2016). For instance, Iran launched an adopted version, called IraPEN, with specific targets for prevention and control of non-communicable diseases. On the other hand, “Screen and Treat” strategies are unlikely to have a substantial impact to reduce the diabetes epidemic. Therefore, they should be complemented by population-wide approache*s* for effective diabetes prevention (Barry et al. 2017).

EMR countries are at different stages of prevention and control of diabetes; all high-income countries except Oman (Bahrain, Kuwait, Qatar, Saudi Arabia, and United Arab Emirates), some of the middle-income EMR countries (Iran, Jordan, Lebanon, and Tunisia), and none of the low-income EMR countries have an operational policy, strategy, or plan of action for diabetes (WHO 2017).

CKD mortality increased in recent years in the region, underscoring the need for better treatment and management of blood pressure and diabetes. Diagnosis and control of diabetes and blood pressure are not optimal in the region. Early detection through screening of high-risk individuals is crucial to control blood pressure and diabetes and reduce diabetes and CKD burden and mortality. Although evidence is not strong enough to conclude that early diagnosis of diabetes will increase survival, treatment of impaired glucose tolerance or impaired fasting glucose, as well as lifestyle interventions, is associated with delayed progression to diabetes (Selph et al. 2015a, b). There is a need for more aggressive programs to control blood pressure and diabetes that include medical and preventive care approaches.

Access to and quality of medical care has a major impact on mortality from diabetes and CKD (Alegre-Diaz et al. 2016). Several studies have suggested that proper treatment might reduce complications and improve outcomes. Both diabetes and CKD require patients to adhere to long-term management of the condition (Brunton and Polonsky 2017). Unfortunately, not all the region’s residents have equal access to quality medical care. It is possible that proper management of these conditions varies by county and has led to the observed increases in mortality.

Several studies have shown that obesity has rapidly increased in the region during the time period of this study (Mokdad et al. 2014, 2016). The studies have shown that inadequate physical activity and high body mass index are common in the region (El Bcheraoui et al. 2016). Moreover, dietary factors are among the major risk factors for diabetes and CKD (Moradi-Lakeh et al. 2016b; Yakoob et al. 2016). For example, low intake of whole grains, nuts and seeds, and fruit, and the consumption of processed food and red meats are known risk factor for diabetes; and high sodium intake is an important risk factor for CKD (Afshin et al. 2015; Moradi-Lakeh et al. 2016b). Diet has not improved much in the region during the study period (Afshin et al. 2015; Melaku et al. 2016; Otto et al. 2016). Moreover, there is only limited local information on dietary habits in the region (Afshin et al. 2015; Moradi-Lakeh et al. 2017). There is a need for programs to improve diet and physical activity and to control weight gain in the region to reduce the burden of diabetes as well as many other conditions. Only a few EMR countries have an operational policy, strategy, or plan of action to reduce obesity and physical inactivity (WHO 2017). The countries need to target different age groups, especially youth, to initiate sustainable changes in lifestyle. High intake of processed meat, sugar-sweetened beverages, and salt, and low intake of fruits and vegetables and whole grains need to be specifically addressed with regard to obesity, diabetes, and CKD-DM (Mokdad et al. 2016; Moradi-Lakeh et al. 2016a, 2017a; Ng et al. 2014).

Our study has a few limitations. For many countries with sparse data, estimates were driven by covariates in statistical modeling. The attributable effect of high body mass index (BMI) on ischemic heart disease, stroke, and diabetes was derived from prospective observational studies and meta-analyses. Our study does not account for variation within countries. We also do not have adequate data on access to and quality of health care in the region. More details on these limitations have been published elsewhere (Moradi-Lakeh et al. 2016b). On the other hand, we used new data for some countries, such as Saudi Arabia, which changed our estimates compared to GBD 2013 (El Bcheraoui et al. 2014; Moradi-Lakeh et al. 2016b).

### Conclusion

Our study showed a large and increasing burden of diabetes in the region. This burden will increase with aging and growth of the population unless effective programs for control and prevention are put in place. Diabetes is a costly disease and most countries in the region spend a large percentage of their health resources on the disease. The region’s financial and manpower resources are already stretched. Hence, there is an urgency to deal with diabetes and its consequences, and these efforts should be at the forefront of disease prevention and health promotion.

## Electronic supplementary material

Below is the link to the electronic supplementary material.

Supplementary material 1 (XLSX 28 kb)
